# Nonfunctional pancreatic neuroendocrine tumor masked as anemia: A case report

**DOI:** 10.1097/MD.0000000000007441

**Published:** 2017-07-07

**Authors:** Baojin Xu, Yue Wang, Xiaoyan Li, Jie Lin

**Affiliations:** a Department of General Surgery; b Department of Pathology, Cancer Hospital of China Medical University, Liaoning Cancer Hospital and Institute, Shenyang, China.

**Keywords:** anemia, nonfunctional pancreatic neuroendocrine tumor

## Abstract

After a series of clinical relevant examinations. The patient was dignosed as pancreatic tomor in the pancreatic tail accompanied with the symptom of anenmia and dizziness.Until now surgery is the best treatment strategy for pancreatic tumors.So we take a joint multiple organ removal surgery.

Before surgery, the main concerns of patient is whether the operation can relieve the anemia-related symptoms and improve the quality of life.

The patient was dignosed as nonfunctional pancreatic neuroendocrine tumor.

A joint multiple organ removal surgery including pancreaticbody and tail, spleen, part of the stomach wall, left adrenal gland,and portal splenic vein thrombosis and lymphadenectomy were performed on this patient.

After surgery, the concentration of hemoglobin gradually increased and remained stable (88 g/L) on the postoperative day7. Furthermore, complete resolution of the symptom of anemia was achieved on postoperative day 30. There was no recurrence of the tumor or the symptom of anemia during the 3-month follow-up.

We conclude that NF-PNETs can manifest as anemia at the time of diagnosis, and if the tumor is resectable, surgical resection is a safe and curative form of therapy not only for the anemia but also for the original tumor.

## 1 Introduction

Neuroendocrine tumors (NETs) are considered rare neoplasms with an annual incidence of 2.5–5 per 100.000. NETs are distributed in the gastrointestinal system and approximately 1/10 develop in pancreas.^[1–3]^ Pancreatic neuroendocrine tumors (PNETs) are a rare group of neoplasms that arise from multipotent stem cells in the pancreatic ductal epithelium. The overall 5-year survival rate is in the range of 5% to 6%.^[4–6]^ For patients with pancreatic neuroendocrine tumors that have metastasized, prognosis is poor, with a survival of only 1–3 years. PNETs are classified clinically as nonfunctional or functional, based on the properties of the hormones they secrete and their ability to produce a clinical syndrome. In addition, nonfunctional PNETs (NF-PNETs) are more common when campared with functional PNETs (F-PNETs). NF-PNETs can secrete multiple peptides and lead to present a wide variety of clinical manifestations. Herein, we describe an unusual case of pancreatic neuroendocrine tumor, which is primarily manifested as anemia.

## 2 Case report

A 36-year-old man was admitted to our hospital presenting anemia, dizziness, and fatigue for the previous 3 weeks along with weight loss of 5 kg. He had suffered a magnetic resonance imaging and gastroscopy at the local hospital. The MRI indicated a mass in the pancreatic tail and splenomegaly. The gastroscopy reported esophageal and gastric varices. A detailed physical examination revealed abdominal pain, the pale nail, and conjunctiva but no sign of jaundice. Besides the patient had a history of smoking for 20 years, consuming 10 cigarettes per day averagely and a history of drinking for 15 years, consuming 2 bottles of beer per day averagely.

**Figure 1:**
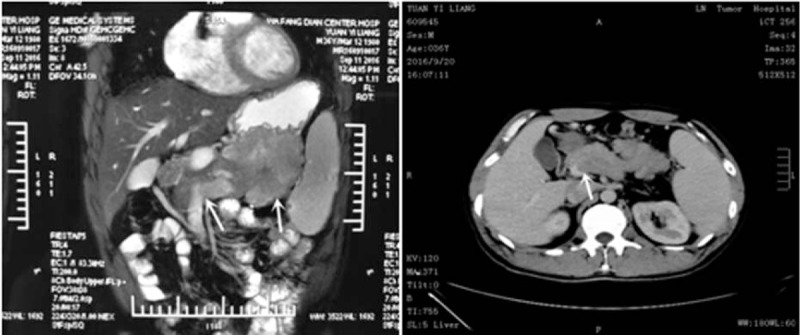
Preoperative MRI and CT scans of our patient indicated a tumor (9.6 cm × 7.6 cm) in the pancreatic tail closely related to the stomach and spleen, and portal vein thrombosis. CT = computed tomography, MRI = magnetic resonance imaging.

An extensive inpatient diagnostic workup was initiated. The concentration of hemoglobin was severely reduced (63 g/L). The white blood cell count and the platelet count was reduced (WBC: 3.45 × 10^9^/L,PLT: 90 × 10^9^/L). In addition, urinalysis, routine stool, and occult blood test were negative. Transaminase and jaundice index were normal. Tumor markers (CEA, AFP, CA19–9) were negative. Abdominal ultrasonography indicated fatty liver, intrahepatic bile duct calcification, splenomegaly, a hypoechoic mass in the pancreatic tail. A computed tomography scan demonstrated splenomegaly, a tumor (9.6 cm × 7.6 cm) in the pancreatic tail closely related to the stomach and spleen, multiple enlarged lymph nodes, and portal vein thrombosis (Fig. 1).

The case was discussed at a multidisciplinary team meeting. It was agreed that surgical resection should be the best treatment. Then, a joint multiple organ removal surgery including pancreatic body and tail, spleen, part of the stomach wall, left adrenal gland, and portal splenic vein thrombosis and lymphadenectomy were performed on this patient.

**Figure 2:**
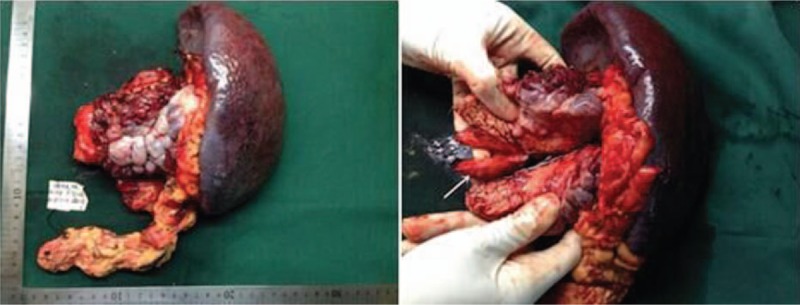
Pathological examination of the resected specimen revealed a mass (9 × 8 × 7 cm) with a cancerous embolus (7 cm) originating from pancreatic and was later diagnosed as the pancreatic neuroendocrine tumor (G3).

**Figure 3:**
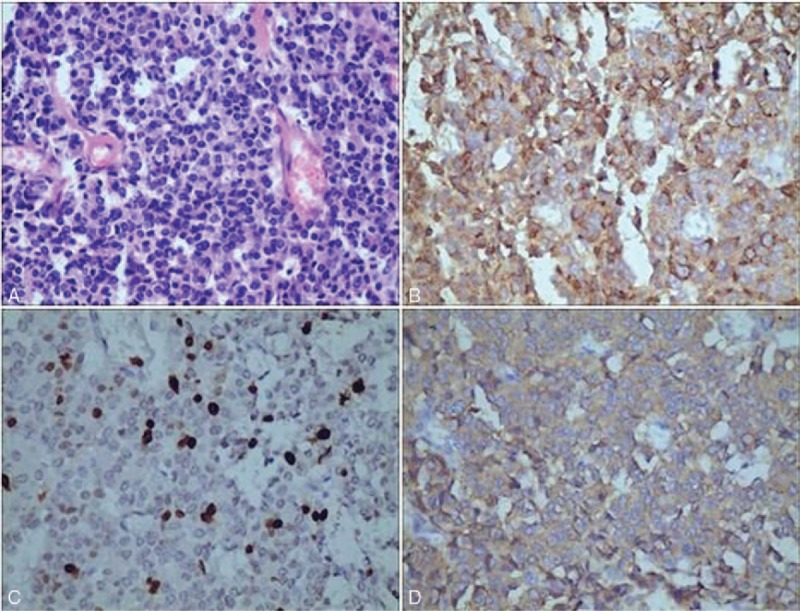
(A) Hematoxylin and eosin staining for the tumor. (B) Immunohistochemistry staining for chromogranin A was positive. (C) Immunohistochemistry staining for Ki-67 was positive. (D) Immunohistochemistry staining for synaptophysin was positive.

Grossly, the resected pancreatic tissue measured 9 × 8 × 7 cm between pancreatic tail and hilum of spleen, with uneven surface, gray and yellowish gray to tan, firm, patical exquisite texture, and solid cut surface (Fig. 2). Hematoxylin and eosin staining of the surgical specimen demonstrated that the mass was composed of many uniform small cells, which was arranged in sheet, cord, or chrysanthemum-like. The cells have granular nuclear chromatin and small distinct nucleoli. The size and shape of these cells were uniform with a high karyoplasmic ratio, and there was round or oval largenuclear lay in the center, granular nuclear chromatin, and small distinct nucleoli. According to these findings, a pancreatic neuroendocrine tumor was considered. Besides the expression of chromogranin A, Ki67 (index≈20%) and synaptophysin was positive (Fig. 3). Consequently, a histopathological diagnosis of pancreatic neuroendocrine tumor (G3) was made.

After surgery, platelet count appeared to be increasing. So the patient received the drug therapy with aspirin (100 mg/d). The concentration of hemoglobin gradually increased and remained stable (88 g/L) on the postoperative day 7. Furthermore, complete resolution of the symptom of anemia was achieved on postoperative day 30. There was no recurrence of the tumor or the symptom of anemia during the 3-month follow-up.

## 3 Discussion

Pancreatic neuroendocrine tumors are different from exocrine tumors of the pancreas (pancreatic adenocarcinoma) not only in their clinical feature but also in their pathology. They are fairly rare and account for approximately 3% of all pancreatic tumors. However, their incidence is increasing, due in part to heightened awareness of the disease, improved diagnostic techniques, and imaging studies.^[7]^

Pancreatic neuroendocrine tumors are divided into 2 major categories: nonfunctional pancreatic neuroendocrine tumors (NF-PNETs) and functional neuroendocrine tumors (F-PNETs). NF-PNETs account for more than 75% of pancreatic neuroendocrine tumors and can synthesize more than 1 peptide to produce all kinds of clinical features. These symptoms may include abdominal pain, weight loss, anorexia, nausea, and jaundice. For this reason, most of NF-PNETs are accidentally discovered, with 70% being greater than 5 cm and more than 60% presenting with synchronous liver metastases.^[4,8]^ F-PNETs are much less common and account for about 20% of pancreatic neuroendocrine tumors. Historically, the 9 commonly recognized F-PNETs include insulinoma, gastrinoma, glucagonoma, VIPoma, somatostatinoma, GRFoma, ACTHoma, PTHrp-oma, and PNET causing carcinoid syndrome.^[9,10]^ They present with specific clinical syndromes related to their hormonal secretions so the clinical diagnosis of F-PNETs are easier than NF-PNETs. Table 1 reviews the characteristics of the commonly recognized PNETs. In addition to these 9, other rare F-PNETs have been described in recent years. The biologically active peptides secreted from rare F-PNETs in the literature include luteinizing hormone,^[11]^ erythropoietin,^[12]^ renin,^[13]^ and pancreatic polypeptide.^[14]^

**Table 1:**
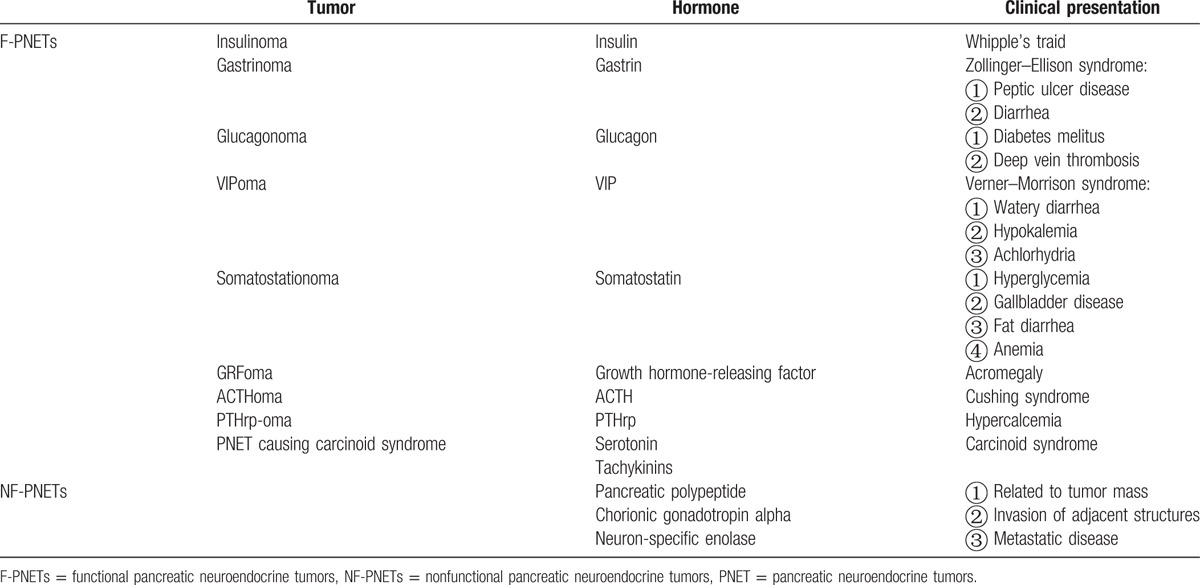
Characteristics of recognized PNETs.

According to the World Health Organization (WHO), anemia is defined as hemoglobin (Hb) levels <12.0 g/dL in women and <13.0 g/dL in men. In terms of development rate, anemia is categorized into 2 classes: acute anemia and chronic anemia. During clinical work, a variety of reasons can lead to anemia, one of the most important reasons is the diseases related to blood system. But underlying causes of anemia also include some chronic diseases, such as infections, cancer, autoimmune, chronic rejection after solid-organ transplantation, and chronic kidney disease.^[15]^ More than 30% of cancer patients have cancer-related anemia (CRA) at the time of diagnosis.^[16]^ Till now, CRA has been described in ovarian cancer, breast cancer, and colorectal cancer.^[16,17]^ Here, we describe an unusual case of nonfunctional pancreatic neuroendocrine tumor masked as anemia.

Anemia is one of the most frequently reported in patients with cancer.^[18]^ The etiopathogenesis mechanism of CRA remains uncertain. One theory is that CRA may be a consequence of chronic inflammation.^[19]^ Pro-inflammatory cytokines including (IL)-6, (TNF)-αinduce changes in the proliferation of erythroid progenitors, erythropoietin production, and survival of circulating erythrocytes. Plasma CRP levels reflect the levels of (IL)-6, which also modulates the concentration and biological activity of hepcidin.^[20]^ Hepcidin strongly induced by IL-6, inhibits duodenal absorption of iron and blocks iron release from macrophages which eventually lead to CRA. Besides, nutrition and metabolic components also induce cancer-related anemia. However, the laboratory assays revealed no evidence of immune response and nutritional change in our patient.

Anemia, esophageal and gastric varices, and splenomegaly are more common in patients with hepatocrirrhosis. But, these syndromes presented in pancreatic neuroendocrine tumors are seldom mentioned. Until now the symptom of anemia is encountered in F-PNETs, usually in glucagonoma and somatostatinoma. Here, we describe an annual case of nonfunctional pancreatic neuroendocrine tumor presenting as anemia. In our case, pancreatic neuroendocrine tumor invaded splenic vein and formed a huge splenic vein thrombosis to obstruct splenic vein reflux, resulting in hypersplenism. This may be the main cause of anemia in the patient. Regional portal hypertension (RPH) is a pathological condition resulting from outflow obstruction of the splenic vein, which creates a high pressure system in the left side of the portal venous system with formation of collaterals and gastroesophageal varices.^[21,22]^ So the patient eventually manifest anemia and gastroesophageal varices.

Nonfunctional pancreatic neuroendocrine tumors (NF-PNETs) have an aggressive ability, compared with pancreatic carcinoma and functional pancreatic neuroendocrine tumors (F-PNETs). Therefore, they usually invade the surrounding organs and blood vessels to produce a series of clinical presentation. What's more, the NF-PNETs are a progressive disease and most of patients are discovered with liver metastases. Due to the low sensitivity of chemotherapy and targeted drugs, surgery is often as the preferred therapy for pancreatic neuroendocrine tumor.

## 4 Conclusion

Finally, we conclude that NF-PNETs can manifest as anemia at the time of diagnosis, and if the tumor is resectable, surgical resection is a safe and curative form of therapy not only for the anemia but also for the original tumor. However, considering some limitations in the case report, a further multicenter study is necessary.
